# Transfemoral transcatheter aortic valve implantation in patients with end-stage renal disease and kidney transplant recipients

**DOI:** 10.1038/s41598-017-14486-7

**Published:** 2017-10-31

**Authors:** Fadi Al-Rashid, Anja Bienholz, Heike Annelie Hildebrandt, Polycarpos-Christos Patsalis, Matthias Totzeck, Andreas Kribben, Daniel Wendt, Heinz Jakob, Alexander Lind, Rolf Alexander Jánosi, Tienush Rassaf, Philipp Kahlert

**Affiliations:** 1 The Department of Cardiology and Vascular Medicine of the West-German Heart and Vascular Center Essen, Essen University Hospital, University Duisburg-Essen, Essen, Germany; 2 The Department of Cardiovascular Surgery of the West-German Heart and Vascular Center Essen, Essen University Hospital, University Duisburg-Essen, Essen, Germany; 3 The Department of Nephrology, Essen University Hospital, University Duisburg-Essen, Essen, Germany

## Abstract

Transcatheter aortic valve implantation (TAVI) has evolved to a treatment of choice in high-risk patients and is therefore ideal for patients with advanced chronic kidney disease, as patients with end-stage renal disease and kidney transplant recipients. Especially, outcome of this special patient group is very important. 22 patients with chronic kidney disease stage 5 undergoing intermittent hemodialysis treatment (CKD 5D) and 8 kidney transplant recipients (KT) with severe aortic valve stenosis underwent transfemoral TAVI. TAVI was successfully performed in all patients. Postinterventional acute kidney injury (AKI) occurred in four kidney transplant recipients (KDIGO grade 1: n = 3, grade 3: n = 1) but creatinine/eGFR returned to baseline values in all patients. Short-term (30-day) mortality was 3% (1 patient in CKD 5D group). KT had a higher 2-year mortality than CKD5D patients (31% vs. 53%; p = 0.309), and cause of death was non-cardiac because of sepsis in all cases. The amount of contrast medium during TAVI was not associated with the development of acute kidney injury. TAVI is feasible in patients with CKD5D and in KT. Postinterventional AKI in these patients is often mild and does not impact renal function at day 30, while infection/ sepsis is the leading cause of mid-term mortality.

## Introduction

Transcatheter aortic valve implantation (TAVI) has evolved to a treatment of choice in high-risk patients with symptomatic aortic valve stenosis. Patients with impaired renal function prior to the procedure, especially patients with end-stage renal disease and kidney transplant recipients, represent a special high-risk subgroup.

The outcome of patients with end-stage renal disease and kidney transplant recipients undergoing TAVI is not well-explored but of interest, since TAVI might be a viable alternative to surgical aortic valve replacement for these patients. In the setting of cardiac surgery a decreased estimated glomerular filtration rate (eGFR) is known as a major risk factor for adverse postoperative outcome^1^, and preoperative chronic hemodialysis reveals high mortality rates after TAVI^2^. Short-term mortality of kidney transplant recipients undergoing valvular heart surgery is 14%^3^, while TAVI seems to be an effective method to treat kidney transplant recipients with reduced short-term mortality^4^. Nevertheless, no data exist on mid-term outcome after TAVI. Besides outcome, a chronic impairment of the kidney transplant is very important. As already stated, acute kidney injury following transcatheter aortic valve implantation (TAVI) is associated with an increased short- (i.e. 30-day) and mid-term mortality^5^.

Here, we evaluate short-term and mid-term mortality as well as development of postinterventional acute kidney injury in patients with renal replacement therapy and in kidney transplant recipients undergoing transfemoral aortic valve replacement.

## Materials and Methods

### Patient Population

We retrospectively analyzed data of 30 patients undergoing transfemoral TAVI. 22 patients had chronic kidney disease stage CKD G5 as defined by Kidney Disease: Improving Global Outcomes (KDIGO) CKD Work Group^6^ and underwent intermittent hemodialysis treatment prior to the procedure (CKD 5D). 8 patients were kidney transplant recipients (KT).

These patients were part of a consecutive cohort of 710 high-risk or inoperable patients with severe symptomatic aortic valve stenosis who underwent transfemoral TAVI at our center between 2006 and 2016. The local ethics committee of the University of Duisburg-Essen approved this retrospective analysis (No. 16-6894-BO). All procedures were performed in accordance with relevant guidelines and regulations. All patients gave written informed consent for study participation and publication, and the study conformed to the principles of the Declaration of Helsinki.

The indication for TAVI in the individual patient was a consensus decision of the multidisciplinary heart-team (consisting of cardiologists, cardiac surgeons, anesthesiologists and physicians from other disciples whenever needed) according to current guidelines^7^.

### TAVI Procedure

TAVI was performed by a multidisciplinary heart-team in a hybrid operating room using standard techniques^8,9^, predominantly under conscious sedation^10^ with percutaneous femoral artery access and closure^11^. One of two currently CE-approved bioprosthesis (Edwards Sapien and Medtronic CoreValve) was implanted.

All patients were periprocedurally monitored with a 6-electrode virtual 12-lead electrocardiogram and pulse oximetry; an indwelling urinary catheter was inserted. A radial artery catheter and a triple lumen central venous catheter in the internal jugular vein (under ultrasound guidance) were placed, along with a pulmonary artery balloon catheter and a provisional pacemaker catheter^10^.

All patients were routinely transferred to the intensive care unit (ICU) after the procedure for postinterventional surveillance and further care for a minimum of 24 hours.

### Definition acute kidney injury

AKI was defined and staged based on serum creatinine analogous to non-transplant patients according to KDIGO^12^. Grade 1 is defined as a serum creatinine increase to 1.5–1.9 times baseline or ≥0.3 mg/dl increase within 48 h, grade 2 as an increase in serum creatinine to 2.0–2.9 times baseline, and grade 3 to a serum creatinine increase to 3.0 times baseline or increase to ≥4.0 mg/dl or initiation of renal replacement therapy.

Pre-interventional (24 h before procedur) serum creatinine was defined as baseline. Serum creatinine values were measured daily in all KT for at least 7 days following TAVI.

### Statistical Analysis

Data are presented as mean ± standard deviation if normally distributed or as median and interquartile range otherwise. Categorical variables are given as frequencies and percentages. Categorical data were compared between groups using χ^2^- or Fisher’s exact test. Continuous variables were compared using the Student t-test for dependent and independent samples or the Mann–Whitney U and Wilcoxon signed-rank tests. Kaplan-Meier survival functions were compared with log-rank test. A p-value < 0.05 was considered significant.

Follow-up included data for at least two years following TAVI in each individual patient. All analyses were performed using PASW [SPSS] (Version 21.0, IBM SPSS, Chicago, IL, USA). The authors had full access to the data and take responsibility for their integrity. All authors have read and agreed to the manuscript as written.

## Results

### Patient characteristics

Our study cohort represents a typical transfemoral TAVI population with severe, symptomatic aortic valve stenosis and high operative risk due to age and comorbidities in addition to renal disease (Table 1). Kidney transplant recipients (KT) were obviously younger (73 ± 4 vs. 79 ± 5 years; p = 0.004) and at lower risk (logistic EuroSCORE 9 ± 5 vs. 27 ± 11%%; p < 0.001) than patients with CKD 5D. Kidney transplant recipients were graded in KDIGO stage CKD 3 T in 3 (38%) and CKD 4 T in 5 cases (62%) at baseline.

**Table 1:** Patient Characteristics.

	All patients n = 30	Kidney transplant recipients n = 8	CKD 5D n = 22	p-value
Age [yrs.], mean ± SD	78 ± 6	73 ± 4	79 ± 5	0.004
Female sex, n (%)	18 (60)	1 (13)	5 (23)	0.067
Logistic EuroSCORE [%], mean ± SD	22 ± 13	9 ± 5	27 ± 11	<0.001
STS Score[%], mean ± SD	11 ± 10	5 ± 3	13 ± 11	0.075
Coronary artery disease, n(%)	18 (60)	4 (50)	11 (50)	0.517
Prior cardiac surgery, n(%)	5 (17)	1 (13)	4 (18)	0.820
Ejection fraction [%], mean ± SD	48 ± 16	54 ± 5	46 ± 17	0.179
Chronic obstructive lung disease, n(%)	6 (20)	1 (13)	5 (23)	0.552
Estimated glomerular filtration rate, mean ± SD		25 ± 7		
Creatinine [mg/dl], mean ± SD	4.2 ± 2.1	2.2 ± 0.7	4.9 ± 1.9	0.002
Pulmonary hypertension, n(%)	10(33)	1 (13)	9 (41)	0.155
NYHA functional class, median (range)	3 (2–4)	3 (2–4)	3 (2–4)	0.856
Aortic valve area [cm^2^], mean ± SD	0.6 ± 0.2	0.7 ± 0.1	0.6 ± 0.2	0.404
Mean transaortic pressure gradient [mmHg], mean ± SD	40 ± 16	49 ± 22	37 ± 13	0.136

### Procedure

TAVI was successfully performed in all patients with CKD 5D and in kidney transplant recipients (Table 2). Vascular complications (KT vs. CKD 5D: 13 vs. 18%; p = 0.820), bleeding (25 vs. 14%; p = 0.275) and postprocedural pacemaker implantation (0 vs. 18%; p = 0.208) did not differ between the groups. The different rate in pacemaker implantations can be expected by the fact that the self-expandable bioprosthesis was used in all cases with CKD 5D.

**Table 2:** Postoperative data.

	All patients n = 30	Kidney transplant recipients n = 8	CKD 5D n = 22	p-value
Edwards Sapien, n(%)	28 (93)	8 (100)	18 (82)	
Medtronic CoreValve, n(%)	4 (13)	0	4 (18)	
Contrast agent [ml], mean ± SD	113 ± 34	116 ± 32	112 ± 35	0.804
Postprocedural AI > = II, n(%)	1 (3)	0	1 (5)	0.912
Pacemaker Implantation, n(%)	4 (13)	0	4 (18)	0.208
Coronary Obstruction, n(%)	1 (3)	0	1 (5)	0.556
Pericardial effusion, n(%)	1 (3)	1 (13)	0	0.181
Stroke, n(%)	0	0	0	
Myocardial Infarction, n(%)	1 (3)	0	1 (5)	0.912
CPR, n(%)	3 (10)	0	3 (14)	0.295
AKI, n(%)		4 (50)		
Bleeding, n(%)	5 (17)	2 (25)	3 (14)	0.275
Vascular Complications, n(%)	5 (17)	1 (13)	4 (18)	0.820

### Acute Kidney Injury in Kidney Transplant Recipients

Postinterventional acute kidney injury occurred in four kidney transplant recipients (50%) (Table 3). Acute kidney injury was classified KDIGO grade 1 in three cases, while a single dialysis treatment was performed in one patient (KDIGO grade 3). Serum creatinine had returned to baseline values in all patients at day 30. The amount of contrast agents used was 113 ± 34 ml, and there was no significant difference between the two groups. It was also not associated with the development of acute kidney injury (“AKI” contrast agent: 115 ± 28 ml vs. “no AKI” contrast agent:113 ± 39, p = 0.915). Renal function recovered at day 7 after TAVI procedure (CKD 2 T n = 1; CKD 3 T n = 3; CKD 4 T n = 4).

**Table 3:** Kidney Injury in KT.

	n = 8
AKI Grade 1, n(%)	3 (38)
AKI Grade 2, n(%)	0
AKI Grade 3, n(%)	1 (13)
Creatinine at Baseline [mg/dl], mean ± SD	2.1 ± 0.8
Creatinine at 24 h [mg/dl], mean ± SD	2.1 ± 1.1
Creatinine at 48 h [mg/dl], mean ± SD	2.5 ± 1.0
Creatinine at 72 h [mg/dl], mean ± SD	2.6 ± 1.0
Creatinine at 7 days [mg/dl], mean ± SD	2.3 ± 0.9
Creatinine at 1 year [mg/dl], mean ± SD	2.3 ± 1.1
eGFR at Baseline, mean ± SD	28 ± 9
eGFR at 24 h, mean ± SD	33 ± 19
eGFR at 48 h, mean ± SD	27 ± 19
eGFR at 72 h, mean ± SD	24 ± 13
eGFR at 7 days, mean ± SD	29 ± 17
eGFR at 1 year, mean ± SD	31 ± 22

### Mortality

**Figure 1:**
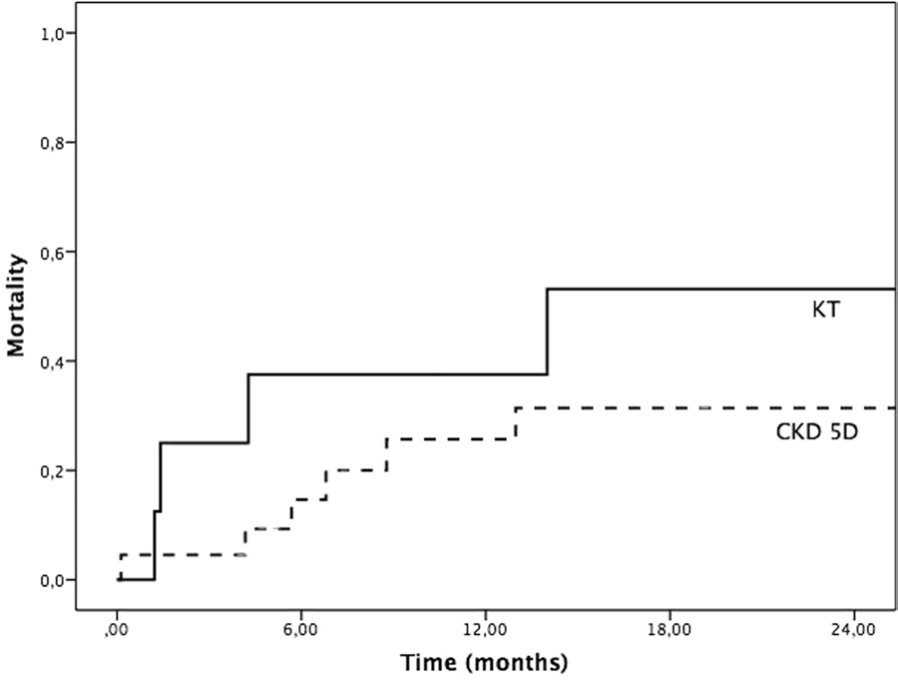
Mid-term mortality. 2-year mortality rate of hemodialysis patients (CKD 5D) and kidney transplant recipients (KT).

Short-term mortality in this special patient cohort was 3% (one patient in the CKD 5D group). One-year (KT vs. CKD5D: 38% vs. 23%; p = 0.409) and 2-year mortality (KT vs. CKD5D: 53 vs. 31%; p = 0.309) (Fig. 1) was higher in kidney transplant recipients compared to the CKD 5D group. If mortality data is compared to “non-kidney” TAVI patients short-term mortality is lower (KT 0% vs. CKD 5D 3% vs. “non-kidney” 8%; p = 0.596), but 1-year (KT 38% vs. CKD 5D 23% vs. “non-kidney” 19%; p = 0.371) and 2-year mortality (KT 53% vs. CKD 5D 31% vs. “non-kidney” 23%; p = 0.188) are higher in this special patient cohort.

### Cause of death

In the period of two years after TAVI, a total of 10 patients died (4 KT and 6 CKD 5D patients). The majority of patients (80%) died due to infections/sepsis (e.g. pneumonia) (Table 4). All 4 deceased kidney transplant recipients died as a result of infections. There were only 2 cardiovascular deaths. One patient (CKD 5D) died at post-interventional day 4 due to systolic heart failure and non-ST elevation myocardial infarction. This patient had a baseline left-ventricular ejection fraction of 11% and was therefore at excessive risk for cardiovascular complications. The other patient died at day 173 due to unknown cause and was therefore classified cardiovascular according to VARC 2 criteria^13^.

**Table 4:** Cause of death.

Survival (days)	Cause of death
**Kidney transplant recipients**	
37	Sepsis due to Candida ssp.
43	Sepsis due to HSV pneumonia and peritonitis after perforation of rectum
130	Urosepsis due to E. coli (3 MRGN)
426	Sepsis
**CKD 5D**	
4	NSTEMI, heart failure
127	Pneumonia
173	Unknown
207	Sepsis due to pneumonia
267	Sepsis
395	Pneumonia

## Discussion

This retrospective analysis of single-center data describes short- and mid-term, 2-year outcomes of end-stage renal disease patients and kidney transplant recipients undergoing transfemoral TAVI with focus on the development of postinterventional acute kidney injury and causes of death. Our data revealed that (i) TAVI is feasible and safe with a low short-term mortality in this per se high-risk patient cohort, (ii) the leading cause of death was infection/sepsis during follow-up time and that (iii) acute kidney injury was mild in the majority of cases.

Transfemoral TAVI has meanwhile become a well-established and standardized procedure with low complication rates. Although end-stage renal disease is taken into account in risk scores, kidney transplant recipients are not reflected but apparently need to be considered high-risk patients as well. Hence, we sought to evaluate these two special patient cohorts.

### Kidney transplant recipients

Data regarding cardiac survival in kidney transplant recipients are limited and reveal high mortality rates about 20% per year in patients receiving valve replacement^3^. Therefore, TAVI might be a safe alternative for this rarely investigated cohort^4^. Yet, TAVI entails certain special risks for kidney transplant recipients. The access site already carries a high level of risk for the transplant, since a local dissection of the pelvic vessels could lead to an impaired blood circulation. The development of AKI, known as an important risk factor for short-term mortality after TAVI in general^14–16^, also compromises transplant function. A postinterventional increase in creatinine with the development of AKI was developed in 50% of the kidney transplant recipients in our study. AKI was mild in 3 of 4 cases and not associated with a higher operative mortality.

In addition, kidney transplant recipients receive immunosuppression therapy, including steroids, mycophenolate and calcineurin inhibitors. Hence, a high short-term mortality might be expected, as TAVI is associated with systemic inflammatory response syndrome (SIRS), which is a strong predictor of mortality^17^. We did not observe any death within 30 days after procedure. Yet, mid-term mortality rates in kidney transplant recipients were higher than in CKD 5D patients, and, interestingly, all four deceased kidney transplant recipients died as a result of an infection.

### CKD 5D patients

Chronic kidney disease (CKD) is an important predictor of mortality after cardiac surgery and has been included in the risk scores in cardiac surgery^18,19^. Different studies on surgical aortic valve replacement in hemodialysis patients have been performed^20,21^ clearly demonstrating an increased surgical risk in these patients. Hemodialysis and severe CKD are also strongly associated with increased mortality^2,22^ in TAVI patients, but early published data are contradictory and only based on small patient numbers^2,23^. More Recent data revealed a high short- and mid-term mortality in patients with advanced CKD (stage 4 and 5)^24^.

TAVI intuitively appears as a reasonable option in these high-risk patients. However, they are often judged as too sick even for TAVI. Our study showed a low (5%) short-term mortality, but an unexpectedly high mid-term mortality of 31%. The mid-term mortality is comparable to recent published Italian data by Conrotto *et al*. (CKD 5D 2-year mortality 56%)^24^. The higher mid-term mortality of the Italian data could be associated with the higher rate of transapical TAVI (51%)^24^, which is known to be associated with higher mortality rates in contrast to transfemoral TAVI^25,26^. Interestingly, the majority of hemodialysis patients died due to infections during the follow-up period. Our experiences show that TAVI is feasible and safe in hemodialysis patients, whereas open surgery is still associated with a substantial rate of mortality up to 20.7% after surgery^21^. In special cases TAVI was already preferred instead of surgical aortic valve replacement in younger high-risk dialysis patient waitlisted for kidney transplantation due to existing comorbidities^27^. The uncertainty about the use of TAVI in this population can only be clarified by a dedicated trial.

### Limitations

This is a single-center, retrospective observational report with methodology-inherent potential bias that is common for these types of studies. Patients were treated with TAVI over a long time period. Thus, refinements in the TAVI procedure, and also in surgical valves, are not accounted for. Due to this special patient cohort, our study consists of a small number of patients (4.2% of the overall cohort), which only leads to a hypothesis-generating conclusion.

## Conclusions

TAVI is feasible and safe in patients with CKD5D and in kidney transplant recipients, who would not be considered candidates for conventional aortic valve replacement due to their high burden of comorbidities. Postinterventional acute kidney injury in these patients is present, but often mild and does not impact renal function at day 30. Infection/Sepsis is the leading cause of mid-term mortality.
